# Pulmonary Function, Computed Tomography Lung Abnormalities, and Small Airway Disease after COVID-19: 3-, 6-, and 9-Month Follow-Up

**DOI:** 10.3390/jcm13102733

**Published:** 2024-05-07

**Authors:** Krzysztof Kłos, Dominika Jaskóła-Polkowska, Katarzyna Plewka-Barcik, Renata Rożyńska, Ewa Pietruszka-Wałęka, Magdalena Żabicka, Marta Kania-Pudło, Artur Maliborski, Katarzyna Plicht, Grzegorz Angielski, Andrzej Wojtyszek, Karina Jahnz-Różyk, Andrzej Chciałowski

**Affiliations:** 1 Department of Internal Medicine, Infectious Diseases and Allergology, Military Institute of Medicine—National Research Institute, Szaserow Str. 128, 04-141 Warsaw, Poland; kklos@wim.mil.pl (K.K.); kplewka@wim.mil.pl (K.P.-B.); achcialowski@wim.mil.pl (A.C.); 2 Department of Internal Medicine, Allergology, Pneumonology and Clinical Immunology, Military Institute of Medicine—National Research Institute, Szaserow Str. 128, 04-141 Warsaw, Poland; rrozynska@wim.mil.pl (R.R.); kjrozyk@wim.mil.pl (K.J.-R.); 3 Department of Radiology, Military Institute of Medicine—National Research Institute, Szaserow Str. 128, 04-141 Warsaw, Poland; mzabicka@wim.mil.pl (M.Ż.); mkania-pudlo@wim.mil.pl (M.K.-P.); amaliborski@wim.mil.pl (A.M.); 4 7th Polish Navy Hospital, Polanki Str. 117, 80-305 Gdansk, Poland; k.plicht@7szmw.pl (K.P.); grzegorzangielski1@gmail.com (G.A.); a.wojtyszek@7szmw.pl (A.W.)

**Keywords:** SARS-CoV-2, COVID-19 lung function, chest CT abnormalities, small airway

## Abstract

**Background/Objectives:** Coronavirus disease 2019 (COVID-19) course may differ among individuals—in particular, those with comorbidities may have severe pneumonia, requiring oxygen supplementation or mechanical ventilation. Post-COVID-19 long-term structural changes in imaging studies can contribute to persistent respiratory disturbance. This study aimed to investigate COVID-19 sequels affecting the possibility of persistent structural lung tissue abnormalities and their influence on the respiratory function of peripheral airways and gas transfer. **Methods:** Patients were divided into two groups according to severity grades described by the World Health Organization. Among the 176 hospitalized patients were 154 patients with mask oxygen supplementation and 22 patients with high-flow nasal cannula (HFNC) or mechanical ventilation. All tests were performed at 3, 6, and 9 months post-hospitalization. **Results:** Patients in the severe/critical group had lower lung volumes in FVC, FVC%, FEV1, FEV1%, LC, TLC%, and DLCO% at three months post-hospitalization. At 6 and 9 months, neither group had significant FVC and FEV1 value improvements. The MEF 25–75 values were not significantly higher in the mild/moderate group than in the severe/critical group at three months. There were weak significant correlations between FVC and FEV1, MEF50, MEF 75, plethysmography TLC, disturbances in DLCO, and total CT abnormalities in the severe/critical group at three months. In a mild/moderate group, there was a significant negative correlation between the spirometry, plethysmography parameters, and CT lesions in all periods. **Conclusions:** Persistent respiratory symptoms post-COVID-19 can result from fibrotic lung parenchyma and post-infectious stenotic small airway changes not visible in CT, probably due to persistent inflammation.

## 1. Introduction

Coronavirus disease 2019 (COVID-19) is caused by Severe Acute Respiratory Syndrome Coronavirus 2 (SARS-CoV-2), an RNA virus. The SARS-CoV-2 pandemic initially started as an epidemic in Wuhan, a city in China, and the original strain of the virus was called Wuhan-Hu-1 [1]. The genetic sequence of the Wuhan-Hu-1 strain was 80% the same as the sequence of the virus called SARS-CoV that was responsible for Severe Acute Respiratory Syndrome (SARS) in Asia in the years 2002 and 2003 and Middle East Respiratory Syndrome (MERS) in the 2012 in Saudi Arabia [2]. The disease is characterized by high infectiousness and mortality and is transmitted mainly by droplets to the respiratory system, seriously threatening the safety and health of people [3].

COVID-19 is a heterogeneous disease, meaning the clinical course may proceed differently, and the prognosis significantly differs between individuals. Most of them experience asymptomatic or minimally symptomatic infection, and spontaneous recovery is expected within this group. The most common symptoms are fever, dry cough, and fatigue, and in some people, variously expressed shortness of breath or dyspnea occurs. However, some individuals, especially those with other comorbidities, may have a severe disease course, with pneumonia requiring treatment in the inpatient care unit and oxygen therapy supplementation or mechanical ventilation due to acute respiratory distress syndrome (ARDS) [4].

Approximately one-third of COVID-19 cases were associated with ARDS and severe pulmonary complications, such as the development of pulmonary fibrosis or other abnormalities of the lung parenchyma, which has sustained character in approximately 30% of individuals after 3 and 6 months post-recovery. It was shown that the development of persistent lung parenchyma abnormalities was related to the duration of the acute infection, the severity of the disease, and patients’ general conditions. There were risk factors associated with a more severe clinical course, such as older age or male sex [5].

Post-COVID-19, long-term pulmonary outcomes are still not fully understood. Some studies, for instance by Han and Wu et al. [5,6], describe pulmonary sequelae in COVID-19 survivors after a few months (4 or 6 months or longer) after the resolution of the acute illness. However, according to follow-up studies, more extended time is necessary, as we believe that in some individuals, the structural changes in X-ray examination or, more often, computed tomography (CT) and pulmonary function tests (PFT)—restrictive or obstructive or diffusion limitation disturbances in gas transfer—may be persistent [4,5,6]. This study aimed to investigate COVID-19 sequels affecting the possibility of persistent structural lung tissue abnormalities and their influence on respiratory function in peripheral airways and gas transfer, as well as determine the differences in outcomes between individuals who experienced mild/moderate and severe/critical disease courses.

## 2. Materials and Methods

This study, which was conducted retrospectively, included patients with different courses of COVID-19 who were hospitalized in the Department of Internal Medicine, Infectious Diseases, and Allergology, Military Institute of Medicine in Warsaw, and the 7th Polish Navy Hospital between November 2020 and April 2022.

The inclusion criteria for participants included an age above 18 years old, a positive test for novel coronavirus via real-time PCR at the time of admission, available initial chest CT scans with typical lung COVID-19 lesions, and being able and willing to give informed consent to take part in this study. The exclusion criteria included pregnancy, lung cancer, tuberculosis or a history of lung surgery, other lower respiratory tract infections, i.e., influenza, and insufficient quality of CT scan image.

Patients were divided into two groups according to severity grades described by the World Health Organization (WHO): 1. mild or moderate disease with clinical signs of pneumonia and peripheral oxygen saturation (SpO_2_) >90% (mild/moderate); 2. severe disease with pneumonia and SpO_2_ < 90%, respiratory rates > 30 breaths/min, or critical disease, i.e., progression of pulmonary imaging lesions > 50% within 24–48 h, ARDS, sepsis, or septic shock (severe/critical).

The Military Institute of Medicine Ethics Committee 3/WIM/2021 of 20 February 2021 approved this study, and all enrolled patients provided written informed consent. All patients underwent lung CT scanning at hospital admission. The pulmonary function tests (PFT) were not performed because of active acute SARS-CoV-2. After 3, 6, and 9 months of hospital discharge, examinations were conducted and they consisted of PFT–spirometry, plethysmography, and diffusion capacity for carbon monoxide (DLCO), as well as chest CT scans. All patients in 3 periods—at baseline and 6 and 9 months after discharge—underwent a pulmonary function test (PFT) following the 2017 American Thoracic Society (ATS) and European Respiratory Society (ERS) lung function guidelines [7,8]. The same technician performed all tests using a JAEGER MasterScreen Body/Diffusion device (~230V, 50/60Hz, 508VA, IP20). Lung function parameters included forced vital capacity (FVC); forced expiratory flow in the first second (FEV1); the ratio of forced expiratory flow in the first second to forced vital capacity (FEV1/FVC); the maximal expiratory flow rates at 25%, 50%, and 75% (MEF 25–75) of forced vital capacity; total lung capacity (TLC); residual volume (RV); the ratio of residual volume to total lung capacity (RV/TLC); and the diffusion capacity for carbon monoxide (DLCO) were measured. These parameters (except DLCO) were expressed as absolute values (volumes in liters and flow in liters/minute) and as the percentage of measured values to predicted values. DLCO was shown as mmol/min*kPa and the percentage of measured values to predicted values [9]. The criteria of lung function were assessed as outlined below: FVC and TLC < 80%—restrictive ventilation disorders; FEV1/FVC < 70%—obstructive ventilation dysfunction; MEF 25–75 < 80%—small airway airflow disturbance; DLCO < 80%—diffuse abnormalities. All participants in this follow-up study received an initial chest CT scan at admission to hospital, which was later compared with CT scans received at the follow-up visits after discharge (Table 3). The initial and follow-up unenhanced chest CT examinations were performed on one of two 64-section scanners: SOMATOM goTOP (SIEMENS) and GE Revolution Evo (GE Healthcare) in the supine position, from the apex to base of lungs, with breath-hold in a deep breath, with the following parameters: 1.5–2.5 mm thick sections, 80–100 kVp, 120–400 mA, lung kernel reconstruction of 1–1.25 mm, and a dose-length product of 89–620 mGy/cm.

The follow-up unenhanced CT examinations were performed on one of two 64-section scanners: Discovery CT 750 HD (GE Healthcare) and GE Revolution Evo (GE Healthcare) in the supine position, from the apex to the base of the lungs, with breath-hold in a deep breath, with the following parameters: 2.5 mm thick sections, 80–120 kVp, 120–400 mA, lung kernel reconstruction of 1.25 mm, and a dose-length product of 302–893 mGy/cm. All follow-up examinations of the specific participant were performed on the scanner used in the first follow-up procedure.

The main CT characteristics, such as the presence of consolidation or ground-glass opacity, honeycombing, reticulations, architectural distortion, and the distribution of the lesions, were later described by two experienced chest radiologists blinded to the clinical statuses of the patients. Pattern distribution was recorded as a semi-quantitative estimation of the CT lung score via the CCTS—the Chest CT score [10]. It included the sum of grades in three lobes of the right lung (upper, middle, and lower) and two in the left lung (upper with lingula and lower), based on a five-point scale (0:0%): 1: <5%; 2: 5–25%; 3: 26–50%; 4: 51–75%; 5: > 75%; range of 0–5 for a lobe and a global scale of 0–25 were used.

The number of follow-up visits at 3, 6, and 9 months after discharge depended on the extent of the participants’ lung involvement at a particular follow-up point with the sum of grades (global scale) 5 as a cut-off value. Summary characteristics are presented as means and standard deviations for continuous parameters. For categorical parameters, frequencies and percentages are presented. The normality of the distribution of analyzed parameters was checked using the Shapiro–Wilk test. As common deviations from a normal distribution were found, it was decided to assess the statistical significance of differences between WHO 4–5 and 6–7 groups using a non-parametric Wilcoxon rank-sum test. The differences between WHO 4–5 and 6–7 groups concerning categorical variables were analyzed using a chi-square or Fisher exact test. The dependence between respiratory parameters and CT results was described using the Spearman correlation coefficient and its significance test. In all analyses, the significance level of alpha = 0.05 was set. Due to the exploratory nature of the analyses, no multiple testing procedures were employed. Analysis was performed using R 4.3.1 statistical software (R Core Team (2023). R: A Language and Environment for Statistical Computing. R Foundation for Statistical Computing, Vienna, Austria. Available online: https://www.R-project.org (accessed on 1 March 2023)).

## 3. Results

Among 176 admitted hospital patients, there were 154 mild/moderate patients (WHO 4–5 scale) with passive nasal cannula oxygen supplementation and 22 severe/critical patients (WHO 6–7 scale) demanding one of the other methods—high-flow nasal cannula (HFNC) ventilation was used for 19 persons (86.4%) and mechanical ventilation was used in 3 cases (13.6%). The patients’ characteristics are shown in Table 1. The mild/moderate patient group consisted of 97 males and 57 females with an age of mean ± SD 57.5 ± 12.4 years, and the severe/critical group, with 14 males and 8 females, had an age of 56.9 ± 10.9 years, with a similar body mass index (BMI). Most patients in both groups have arterial hypertension, asthma, coronary artery diseases, and diabetes mellitus type 2. Patients with the WHO 4–5 scale showed statistically significantly higher oxygenation measured with a pulse oximeter (SpO_2_) (89.8 ± 5.8%) than with the WHO 6–7 scale (81.8 ± 6.2%), and the time to hospital discharge was shorter at 13 ± 7 days versus 24 ± 7 days.

Pulmonary function tests, as mentioned earlier, were performed in the third month after hospital discharge. Patients with severe/critical COVID-19 generally had lower lung volumes in FVC, FVC%, FEV1, FEV1%, TLC, TLC%, and DLCO%, but only in the values of FVC, TLC, TLC%, and DLCO% were there observed statistically significance differences (Table 2). Furthermore, COVID-19 patients with a mild/moderate disease course had a lower ratio of FEV1/FVC than those with severe/critical illness. Six and nine months later, there was no significant FVC and FEV1 value improvement in either group, but this was not true in TLC and DLCO. The MEF 25–75 values, indicating small airway airflow, were not significantly higher in the mild/moderate group than in the severe/critical group after three months post-hospital discharge. After a temporary insignificant improvement, in the sixth month of this study, their lower values were recorded in both groups after nine months compared to the previous period (Table 2). The inference conducted relies on results without employing multiple testing correction, and the authors acknowledge this; therefore, general trends are presented, which appear to be consistent.

In the preliminary examination, both groups of patients differed significantly (*p* = 0.002) in terms of pulmonary lesions. The sum of grades in the mild/moderate COVID-19 group estimates is 11.6 ± 4.0 points, and in the severe/critical group, it is 15.1 ± 4.5 points. In the severe/critical group, 2 patients (8%) presented lesions with a severity of 20–25 points, 11 patients (46%) presented lesions with a severity of 15–20 points, 5 patients (21%) presented lesions with a severity of 10–15 points, and six patients (25%) presented lesions with an intensity of 5–10 points. There were no patients with mild changes (0–5 points). In the mild/moderate group, 79 patients (49%) presented lesions with an intensity of 10–15 points, while there were no patients with lesions with an intensity of 20–25 points, and in 13 patients (9%), the intensity of lesions in the lungs did not exceed 5 points.

Three months after hospital discharge, a follow-up examination was performed on 19 patients with severe disease and 130 with mild disease. The severity of changes decreased in both study groups. Among the severe patients, seven patients (37%) presented lesions with intensities of up to 5 points and 5–10 points, four patients (21%) presented 10–15 points, and one patient (5%) presented in the range of 15–20 points. In the group of mildly ill patients, 65 patients (50%) presented with lesions of up to 5 points, 49 patients (38%) presented with lesions of 5–10 points, 15 patients (12%) presented with lesions of 10–15 points, and one patient (1%) presented with an intensity of 15–20 points. No patients presented changes with an intensity of 20–25 points.

Examinations after six months were performed in 16 persons with severe disease and 117 persons with mild disease. In both groups, the number of people with the severity of lesions of 10–15 points decreased; in the severe group, it decreased from 4 to 2 patients (12%); and in the mild group, it decreased from 15 to 4 patients (3%). In both groups, one patient (6% and 1%, respectively) continued to present changes with an intensity of 15–20 points.

Examinations after nine months were performed in 15 persons with severe disease and 115 with mild disease. The values of the severity of changes were similar to those obtained in this study after six months. In the severe group, 2 patients (13%) presented severity lesions of 10–15 points and 1 patient (6.7%) presented severity lesions of 15–20 points. In the mild/moderate group, 4 patients (3.5%) continued to present changes with an intensity of 10–15 points and 1 patient (0.9%) with an intensity of 15–20 points (Table 3).

**Figure 1:**
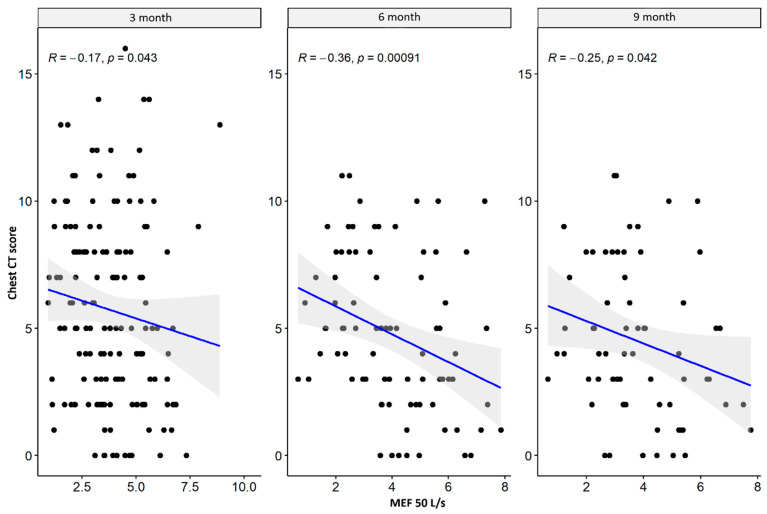
Correlation between MEF 50 and lung CT abnormalities in measurement periods in the mild/moderate patient group.

**Figure 2:**
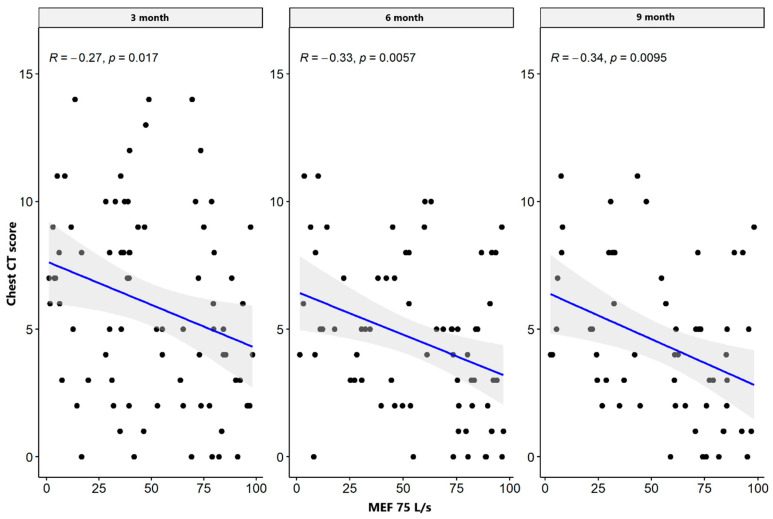
Correlation between MEF 75 and lung CT abnormalities in measurement periods in the mild/moderate patient group.

**Figure 3:**
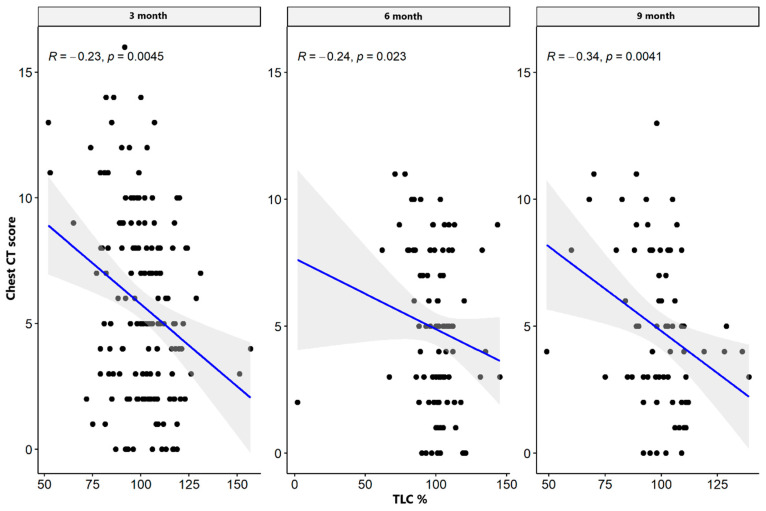
Correlation between TLC% and lung CT abnormalities in measurement periods in the mild/moderate patient group.

**Figure 4:**
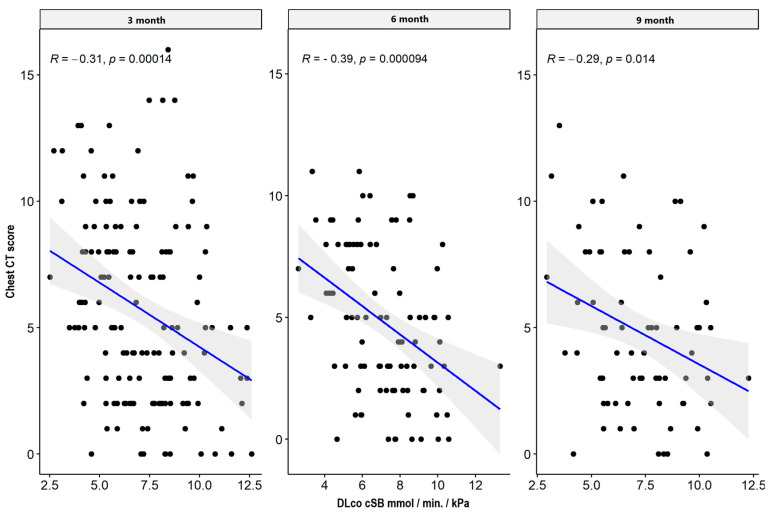
Correlation between DLCO and lung CT abnormalities in measurement periods in the mild/moderate patient group.

**Figure 5:**
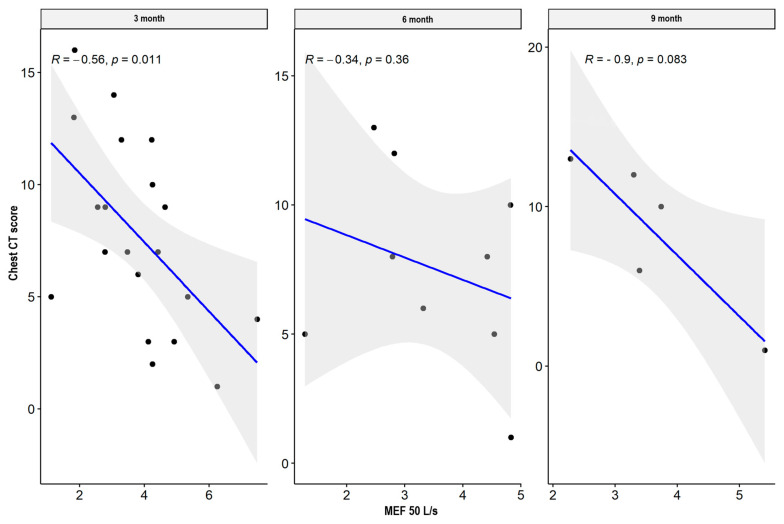
Correlation between MEF 50 and lung CT abnormalities in measurement periods in the severe/critical patient group.

**Figure 6:**
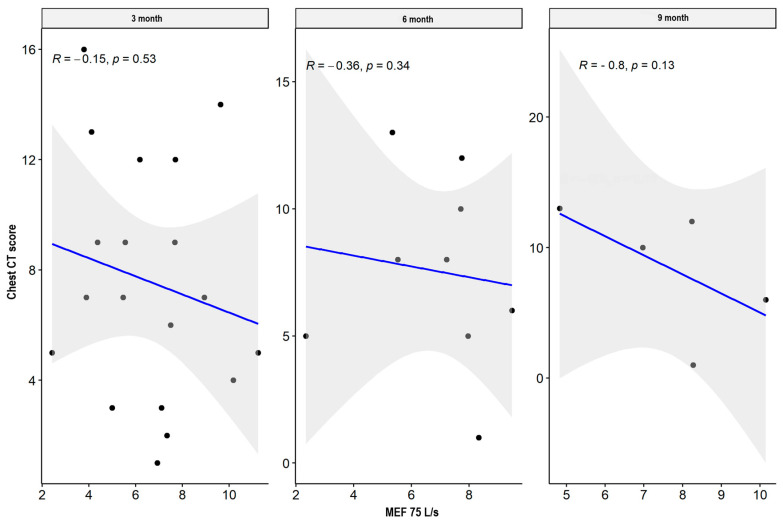
Correlation between MEF 75 and lung CT abnormalities in measurement periods in the severe/critical patient group.

**Figure 7:**
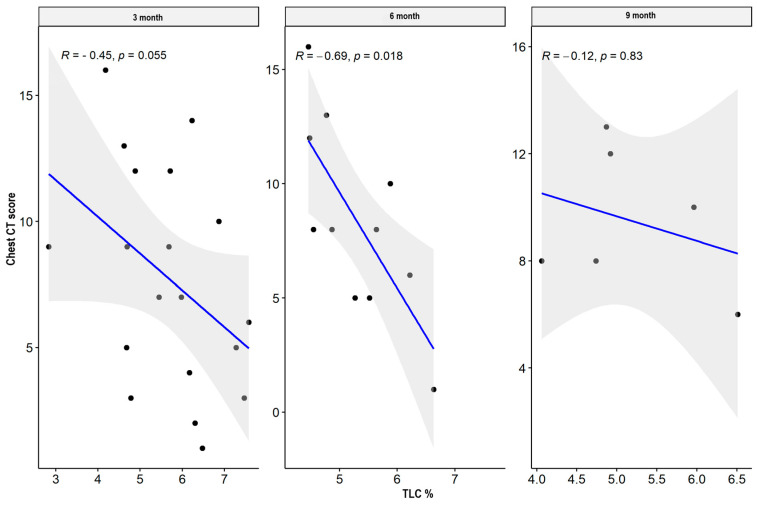
Correlation between TLC% and lung CT abnormalities in measurement periods in the severe/critical patient group.

**Figure 8:**
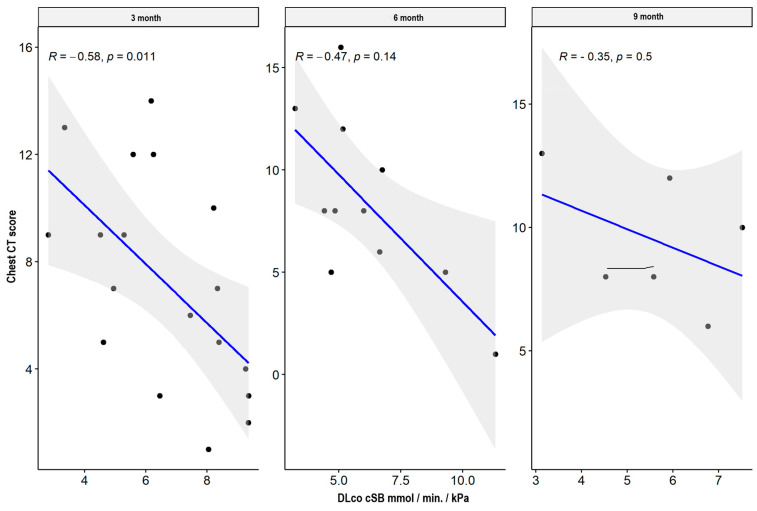
Correlation between DLCO and lung CT abnormalities in measurement periods in the severe/critical patient group.

There were weak significant negative correlations between spirometry values: FVC and FEV1 parameters supported the impairment of airflow through the small airway, while MEF50, MEF 75, plethysmography TLC, disturbances in diffusion capacity for carbon monoxide (DLCO), and the total sum of CT abnormalities in the severe/critical group worsened only three months after hospital discharge. In the mild/moderate group, there was also a significant negative correlation between the spirometry and plethysmography parameters and the total sum of CT lesions in all measurement periods. The results are presented in Table 4 and Table 5 and Figure 1, Figure 2, Figure 3, Figure 4, Figure 5, Figure 6, Figure 7 and Figure 8.

Figures and Tables

**Table 1:** Assessment of inflammatory changes in chest CT score (CTSS) in severe/critical and mild/moderate patients.

Patients		CCTS Score [Points]					
		(0–5)	(5–10)	(10–15)	(15–20)	(20–25)	*p*-Value ^1^
Month 0	Severe/critical *n* = 19	0 (0%)	6 (32%)	4 (21%)	8 (42%)	1 (5.3%)	0.005
	Mild/moderate *n* = 152	13 (8.6%)	43 (28%)	71 (47%)	25 (16%)	0	
Month 3	Severe/critical *n* = 19	7 (37%)	7 (37%)	4 (21%)	1 (5.3%)	0	0.2
	Mild/moderate *n* = 130	65 (50%)	49 (38%)	15 (12%)	1 (0.8%)	0	
Month 6	Severe/critical *n* = 16	6 (38%)	7 (44%)	2 (12%)	1 (6.2%)	0	0.054
	Mild/moderate *n* = 117	71 (61%)	41 (35%)	4 (2.4%)	1 (0.9%)	0	
Month 9	Severe/critical *n* = 15	6 (40%)	6 (40%)	2 (13%)	1 (6.7%)	0	0.042
	Mild/moderate *n* = 115	76 (66%)	34 (30%)	4 (3.5%)	1 (0.9%)	0	

^1^ Fisher exact test. A *p*-value <0.05 was considered statistically significant.

**Table 2:** Baseline characteristics of mild/moderate (WHO 4–5) and severe/critical (WHO 6–7) COVID-19 patients.

Baseline Characteristic	All Patients (*n* = 176)	Disease Severity (WHO Ordinary Scale)		*p*-Value WHO 4–5 vs. WHO 6–7
		Patients WHO 4–5 (*n* = 154)	Patients WHO 6–7 (*n* = 22)	
Sex: male/female *n*/*n*	111/65	97/57	14/8	
Age: years ± SD	56.8 ± 13.6	57.5 ± 12.4	56.9 ± 10.9	0.42 ^1^
Body mass index: kg/m^2^ ± SD	30.0 ± 6.2	30.0 ± 5.8	30.4 ± 4.0	0.73 ^1^
HRCT lung involvement point scale ± SD	12.1 ± 4.2	11.6 ± 4	15.1 ± 4.5	0.42 ^1^
D-dimer: µg/L^−1^ ± SD	1040 ± 1020	1020 ± 990	1160 ± 1230	0.27 ^2^
Mechanical ventilation: *n*/%		0/0	3/13.6	
HFNO: *n*/%		0/0	19/86.4	
Day to hospital discharge: day ± SD	14 ± 8.0	13 ± 7	24 ± 7	0.001 ^1,^*
Oxygenation (SpO_2_): % ± SD	88.7 ± 6.4	89.8 ± 5.8	81.8 ± 6.2	0.001 ^1,^*
Coexisting disorder				
COPD: *n*/%	6/3.4	5/3.2	1/4.5	0.71 ^2^
Asthma: *n*/%	22/12.5	18/11.7	4/18.2	0.76 ^2^
Arterial hypertension: *n*/%	91/51.7	81/52.6	10/45.5	0.53 ^2^
Coronary artery diseases: *n*/%	18/10.2	17/11.0	1/4.5	0.35 ^2^
Chronic heart failure: *n*/%	8/4.5	6/3.9	2/9.1	0.28 ^2^
Atrial fibrillation: *n*/%	13/7.4	13/8.4	0/0	0.17 ^2^
Chronic kidney failure: *n*/%	8/4.5	7/4.5	1/4.5	>0.999 ^2^
Diabetes mellitus type 2: *n*/%	37/21.0	31/20.1	6/27.3	0.44 ^2^
Rheumatoid arthritis: *n*/%	1/0.6	1/0.6	0/0	0.71 ^2^
Pulmonary function at third month of follow-up	value ± SD			
FVC L	3.98 ± 1.24	4.06 ± 1.25	3.49 ± 1.04	0.03 ^1,^*
FVC%	98.73 ± 17.27	99.96 ± 17.34	90.14 ± 14.34	0.39 ^1^
FEV_1_ L	3.14 ± 1.03	3.18 ± 1.05	2.87 ± 0.78	0.12 ^1^
FEV_1_%	97.81 ± 18.63	98.62 ± 19.04	92.18 ± 14.62	0.07 ^1^
FEV_1_/FVC index	78.66 ± 7.91	78.12 ± 7.97	82.39 ± 6.5	0.009 ^1,^*
MEF 75 L/s	6.53 ± 2.3	6.51 ± 2.31	6.63 ± 2.3	0.82 ^1^
MEF 75%	51.07 ± 29.7	50.31 ± 29.85	61.52 ± 27.81	0.38 ^1^
MEF 50 L/s	3.86 ± 1.67	3.86 ± 1.67	3.93 ± 1.49	0.84 ^1^
MEF 50%	108.92 ± 43.25	110.66 ± 43.75	96.75 ± 38.32	0.129 ^1^
MEF 25 L/s	1.2 ± 0.69	1.19 ± 0.7	1.3 ± 0.62	0.43 ^1^
MEF 25%	58.48 ± 30.17	58.9 ± 31.1	51.7 ± 10.96	0.22 ^1^
TLC L	6.32 ± 1.47	6.42 ± 1.47	5.61 ± 1.29	0.01 ^1,^*
TLC%	99.54 ± 15.87	100.99 ± 28.42	89.1 ± 14.97	0.002 ^1,^*
DLCO mmol/min * kPA	7.03 ± 2.21	7.10 ± 2.23	6.45 ± 2.01	0.18 ^1^
DLCO%	78.3 ± 17.18	79.46 ± 17.14	69.51 ± 15.19	0.01 ^1,^*

Note: HRCT: high-resolution computed tomography. HFNO: high-flow nasal cannula. SpO_2_: the saturation of oxygen in the peripheral blood. COPD: chronic obstructive pulmonary disease. FVC: forced vital capacity. FEV_1_: forced expiratory volume in 1 s. FEV_1_/FVC: modified Tiffeneau–Pinelli index. MEF 75: maximal expiratory flow rates at 75% of forced vital capacity. MEF 50: maximal expiratory flow rates at 50% of forced vital capacity. MEF 25: maximal expiratory flow rates at 25% of forced vital capacity. TLC: total lung capacity. DLCO: diffusion capacity for carbon monoxide. ^1^ Mann–Whitney test. ^2^ Chi-square/Fisher exact test. * A *p*-value <0.05 was considered statistically significant.

**Table 3:** Pulmonary function test and diffusion capacity for carbon monoxide values in analyzed patients group and observation periods.

Point of Follow-Up	Three Months			Six Months			Nine Months		
Disease Sevrity	WHO 4–5	WHO 6–7	*p*-Value ^1^	WHO 4–5	WHO 6–7	*p*-Value ^1^	WHO 4–5	WHO 6–7	*p*-Value ^1^
HRCT lung involvement point scale	5.8 ± 3.7	7.7 ± 4.2	0.37	5.1 ± 3.2	6.7 ± 4.3	0.06	5.6 ± 3.3	7.1 ± 4.5	0.09
Oxygenation (SpO_2_%)	97.4 ± 1.8	97.4 ± 1.5	0.27	97.5 ± 1.5	98.3 ± 0.8	0.30	97.7 ± 1.2	97.8 ± 1.1	0.39
Pulmonary function									
FEV_1_/FVC index	78.12 ± 7.97	82.39 ± 6.5	0.009 *	78.05 ± 7.46	80.47 ± 6.68	0.28	77.97 ± 7.13	77.61± 5.83	0.89
FEV_1_/FVC%	100.21 ± 10.51	106.23 ± 7.18	0.002 *	99.71 ± 9.42	103.36 ± 7.35	0.15	99.45± 8.53	101.0 ± 5.29	0.53
FVC L	4.06 ± 1.25	3.49 ± 1.04	0.03 *	4.13 ± 1.18	3.54 ± 1.03	0.10	4.12 ± 1.27	3.87 ± 0.68	0.46
FVC%.	99.96 ± 17.34	90.14 ± 14.34	0.39	97.9 ± 14.75	88.64 ± 13.28	0.05 *	96.13± 17.03	90.5 ±17.66	0.48
FEV_1_ L	3.18 ± 1.05	2.87 ± 0.78	0.12	3.25 ± 1.03	2.83 ± 0.78	0.13	3.23 ± 1.08	3.01 ± 0.58	0.43
FEV_1_%	98.62 ± 19.04	92.18 ± 14.62	0.07	97.6 ±17.59	89.64 ± 10.71	0.05 *	95.76 ± 19.19	90.5 ±14.57	0.43
MEF 75 L/s	6.51 ± 2.31	6.63 ± 2.3	0.82	6.8 ± 2.41	6.76 ± 2.08	0.95	6.73 ± 2.36	7.46 ± 1.85	0.4
MEF 75%	50.31 ± 29.85	61.52 ± 27.81	0.38	54.79 ± 30.8	63.60 ± 17.79	0.31	53.46 ± 29.88	61.11 ± 33.91	0.69
MEF 50 L/s	3.86 ± 1.67	3.93 ± 1.49	0.84	3.93 ± 1.72	3.64 ± 1.18	0.49	3.75 ± 1.63	3.37 ± 1.19	0.49
MEF 50%	110.66 ± 43.75	96.75 ± 38.32	0.129	125.27 ± 47.01	111.73 ± 36.27	0.28	123.92 ± 46.16	110.83 ± 36.27	0.43
MEF 25 L/s	1.19 ± 0.7	1.3 ± 0.62	0.43	1.2 ± 0.69	1.46 ± 0.53	0.63	1.14 ± 0.68	0.93 ± 0.68	0.49
MEF 25%	58.9 ± 31.1	51.7 ± 10.96	0.22	63.1 ± 29.7	63.37 ± 20.21	0.81	64.1 ± 32.11	52.43 ± 26.61	0.45
TLC L	6.42 ± 1.47	5.61 ± 1.29	0.01 *	6.44 ± 1.38	5.47 ± 1.01	0.005 *	6.34 ± 1.46	5.49 ± 0.96	0.04 *
TLC%	100.99 ± 28.42	89.1 ± 14.97	0.002 *	100.3 ± 17.76	89.25 ± 13.98	0.01 *	98.87 ± 14.78	86.56 ± 12.08	0.02 *
DLCO mmol/min * kPA	7.10 ± 2.23	6.45 ± 2.01	0.18	7.06 ± 2.03	3.23 ± 2.11	0.18	7.16 ± 2.03	5.63 ± 1.35	0.02 *
DLCO%	79.46 ± 17.14	69.51 ± 15.19	0.01 *	79.60 ± 18.13	71.64 ± 14.68	0.08	80.85 ± 15.5	67.0 ± 15.15	0.04 *

Note: HRCT: high-resolution computed tomography. SpO_2_: the saturation of oxygen in the peripheral blood. FVC: forced vital capacity. FEV_1_: forced expiratory volume in 1 s. FEV_1_/FVC: modified Tiffeneau–Pinelli index. MEF 75: maximal expiratory flow rates at 75% of forced vital capacity. MEF 50: maximal expiratory flow rates at 50% of forced vital capacity. MEF 25: maximal expiratory flow rates at 25% of forced vital capacity. TLC: total lung capacity. DLCO: diffusion capacity for carbon monoxide. ^1^ Mann–Whitney test. * A *p*-value <0.05 was considered statistically significant.

**Table 4:** Correlation (r) between CT and respiratory function tests in particular measurement periods in mild/moderate patient group.

Lung Function Parameters	HRCT Lung Involvement Point Scale	Three Months		Six Months		Nine Months	
		r	*p*-Value	r	*p*-Value	r	*p*-Value
FVC L	CT lung score	−0.169	0.041 *	−0.299	0.006 *	−0.164	0.193
FEV_1_ L	CT lung score	−0.166	0.046 *	−0.314	0.004 *	−0.191	0.127
TLC L	CT lung score	−0.142	0.089	−0.258	0.014 *	−0.229	0.055
RV%	CT lung score	−0.103	0.218	−0.154	0.146	−0.298	0.012 *
TLC%	CT lung score	−0.235	0.004 *	−0.238	0.023 *	−0.337	0.004 *
DLCO cSB mmol min * kPa	CT lung score	−0.310	0.000 *	−0.394	0.000 *	−0.290	0.014 *
DLCO cSB%	CT lung score	−0.313	0.000 *	−0.305	0.003 *	−0.223	0.062
MEF 25 L/s	CT lung score	−0.173	0.036 *	−0.297	0.007 *	−0.214	0.087
MEF 50 L/s	CT lung score	−0.167	0.043 *	−0.362	0.001 *	−0.253	0.042 *
MEF 75 L/s	CT lung score	−0.086	0.302	−0.289	0.009 *	−0.260	0.036 *
MEF 25%	CT lung score	0.068	0.557	−0.129	0.289	0.123	0.358
MEF 50%	CT lung score	0.002	0.981	−0.245	0.027 *	−0.048	0.704
MEF 75%	CT lung score	−0.270	0.017 *	−0.329	0.006 *	−0.338	0.010 *

Note: HRCT: high-resolution computed tomography. FVC: forced vital capacity. FEV_1_: forced expiratory volume in 1 s. TLC: total lung capacity. RV: residual volume. DLCO cSB: single-breath diffusion capacity for carbon monoxide corrected for haemoglobin. MEF 75: maximal expiratory flow rates at 75% of forced vital capacity. MEF 50: maximal expiratory flow rates at 50% of forced vital capacity. MEF 25: maximal expiratory flow rates at 25% of forced vital capacity. * A *p*-value < 0.05 was considered statistically significant.

**Table 5:** Correlation (r) between CT and respiratory function tests, in particular the measurement periods in the severe/critical patient group.

Lung Function Parameters	HRCT Lung Involvement Point Scale	Three Months		Six Months		Nine Months	
		r	*p*-Value	r	*p*-Value	r	*p*-Value
FVC L	CT lung score	−0.361	0.118	−0.387	0.304	−1.000	0.017 *
FEV_1_ L	CT lung score	−0.420	0.065	−0.376	0.932	0.600	0.017 *
TLC L	CT lung score	−0.446	0.055	−0.694	0.018 *	−0.116	0.827
RV%	CT lung score	−0.416	0.076	−0.009	0.979	−0.754	0.084
TLC%	CT lung score	−0.313	0.191	−0.171	0.616	−0.779	0.068
DLCO cSB mmol min * kPa	CT lung score	−0.581	0.011 *	−0.474	0.141	−0.348	0.499
DLCO cSB%	CT lung score	−0.537	0.022 *	−0.510	0.109	−0.551	0.257
MEF 25 L/s	CT lung score	−0.586	0.008 *	−0.345	0.364	−0.900	0.083
MEF 50 L/s	CT lung score	−0.556	0.011 *	−0.345	0.364	−0.900	0.083
MEF 75 L/s	CT lung score	−0.154	0.528	−0.361	0.339	−0.800	0.133
MEF 25%	CT lung score	−0.058	0.913	0.500	0.450	0.500	1.000
MEF 50%	CT lung score	−0.251	0.285	0.336	0.376	−0.100	0.950
MEF 75%	CT lung score	0.493	0.321	0.000	1.000	−0.500	1.000

Note: HRCT: high-resolution computed tomography. FVC: forced vital capacity. FEV_1_: forced expiratory volume in 1 s. TLC: total lung capacity. RV: residual volume. DLCO cSB: single-breath diffusion capacity for carbon monoxide corrected for haemoglobin. MEF 75: maximal expiratory flow rates at 75% of forced vital capacity. MEF 50: maximal expiratory flow rates at 50% of forced vital capacity. MEF 25: maximal expiratory flow rates at 25% of forced vital capacity. * A *p*-value < 0.05 was considered statistically significant.

## 4. Discussion

The results of our study indicate that despite the improvement in macroscopic lung images, some small airway and lung parenchyma microscopic abnormalities contribute to airflow and gas transfer impairment over a more lengthy period of 12 months. As has been shown in other publications, in approximately 85% of patients, chest CT changes are observed; in 75% of them, these changes are bilateral, with a peripheral subpleural location, initially visible as “groundglass opacities” or “snowstorm,” which may disappear or consolidate later in the course of the disease in the form of fibrosis, taking on the image of a “honeycomb” [11,12,13,14].

Changes in pulmonary fibrosis contribute to impaired respiratory function and the persistence of abnormalities in imaging tests [15]. Due to the short follow-up period, there are no long-term results for COVID-19 survivors. Based on the observations of imaging and functional tests of lungs in people after a course of SARS or MERS, the features of pulmonary fibrosis and respiratory dysfunction have persisted for many years after the initial infection [16,17]. Older age and male sex were significant risk factors for adverse outcomes and the development of pulmonary fibrosis, correlating with the severity and duration of the acute phase of the disease [18,19].

Previous analyses from various countries include three, four, six, or twelve months of observations, and only a few of them last for twenty-four months, reporting on the persistence of changes or their resolution and the improvement in the image in lung CT examinations and respiratory function in respiratory function tests [19,20,21,22,23]. Research carried out by Ye et al. [24] in the months of the increased incidence of the Wuhan type of SARS-CoV-2 in a group of 80 people at two time points, at discharge from the hospital and after three months, showed that the percentage of abnormalities present in the CT images of the lungs, starting as 97.5% in the acute period and decreasing to 12.5% after three months. Pulmonary function tests revealed the presence of particularly restrictive ventilatory dysfunction, small airway dysfunction, and mixed dysfunction, which was more pronounced in patients with severe disease [25]. While Mo et al. [26] concluded that the new coronavirus did not cause permanent lung damage, Dou et al. [27] showed that in COVID-19 patients, lung tissue inflammation differed from interstitial pulmonary fibrosis pathology. However, these studies were not performed in patients with a severe course of the disease. Gonzales et al. [28] indicated that three months after discharge from hospital, 40% of patients still had pulmonary dysfunction and diffusion impairment due to lung structural abnormalities that were consistent with persistent CT changes. These functional impairments are very commonly found in patients with ARDS in a course of COVID-19 who require a stay in the ICU. Swiss multicenter prospective studies conducted four months after discharge from hospital included two groups of patients—mild/moderate and severe/critical patients, according to the severity classification of the World Health Organization. They showed that four months after SARS-CoV-2 infection, the severe/critical course of the disease was associated with significant functional lung abnormalities (limitations in gas exchange, the impairment of DLCO, and exercise tolerance measured via a 6 min walk test (6 MWT)) and radiological issues, potentially caused by disease of the small airways and lung interstitium [22]. Subsequent analysis by Tarraso et al. [29] carried out 12 months after the onset of the acute form of the disease in persons with severe pneumonia (but not treated with mechanical ventilation) showed persistent lung functional disorders measured based on DLCO diffusion capacity in 33% of patients. At the same time, changes in HRCT occurred only in 5% of patients. Similarly, Han and Chen [23] found a gradual, modest reduction in respiratory symptoms from 30% at six months to 22% at two years and gas transfer abnormalities (DLCO) in one-third of patients, while the percentage of subjects showing radiographic changes decreased from 54% at six months to 39% after two years. This would be consistent with previous observations made during the period of SARS and MERS, where, after five years, the functional impairment of the lungs was observed, and the median of the 6 MWT was 75% of the predicted value [30]. The question, therefore, is are there any other morphological and structural changes that could be responsible for the persistence of ventilation disorders? It should be remembered that in patients undergoing hospitalization, HRCT revealed fibrotic changes that may result from a local, not very strong immune response, affecting the pulmonary alveoli and vascular endothelium. According to Hatabu et al. [31], based on the comparison and analysis of HRCT images taken 60 and 100 days after the onset of the disease, a significant improvement was observed in the degree of opacity and consolidation of ground-glass lesions (GGO), which was not observed regarding reticular lesions that affected physical and respiratory performance, as measured via a 6 MWT. Furthermore, DLCO impairment may result from abnormalities present in the distal small airways during a course of constrictive bronchiolitis with air trapping and secondary reflex vasoconstriction or endothelial damage and secondary microthrombosis of the peri-alveolar capillaries. Both of these mechanisms contribute to ventilation/perfusion disturbances [30].

The results of our study may confirm the involvement of small airways in pulmonary pathology and the persistence of respiratory disorders. The correlation found between the degree of lung involvement in CT and PFT parameters, especially MEF 25–75, TLC, and DLCO in all follow-up periods in the mild/moderate group of patients, supports the presence of the limitation of airflow through small airways, and probably also the presence of air trapping (unfortunately not assessed during this study) and gas transfer disorders. The observed relationship between interstitial changes and TLC and DLCO indicates the persistence of restrictive breathing disorders. This is consistent with the study of Torraso et al. [29], who showed that reduced lung diffusion (DLCO < 80%) persisted in almost 40% of patients one year after acute infection, and radiological changes were identified in almost 23% of patients. As reported by Han and Chen [23], after two years, diffusion abnormalities were also observed—the DLCO was < 75%.

It is only puzzling in our study results why there was a connection between ventilation and diffusion disorders and pulmonary lesions in the mild/moderate group despite small pulmonary lesions. However, no such relationship was observed in the severe/critical group. This could have been influenced by the treatment method. In severe/critical cases, it was more aggressive—high oxygen flow, systemic glucocorticosteroids in high doses, and, in some situations, anti-IL-6 (Tocilizumab) were recorded, which probably contributed to the faster local improvement and resolution of lesions, with a subsequent beneficial effect on respiratory mechanics and gas transfer. The symptoms persisted for longer in the mild/moderate group, probably due to pulmonary lesions that were not visible in the CT scan. They are probably due to the persistence of fibrotic interstitial abnormalities, whereas non-fibrotic ones are more likely to recover even after two years. Therefore, according to van Beek [32], CT is not a completely perfect tool for assessing lung function because in the course of COVID-19—as mentioned earlier—there are also abnormalities in the field of microcirculation, contributing to heterogeneous ventilation and perfusion, which is impossible to assess using thin-section computed tomography. Therefore, other more accurate imaging methods should be used, such as dual-energy CT, enabling the study of perfusion, or hyperpolarized pulmonary xenon 129 (129Xe) MRI scans to assess possible limitations in oxygen transfer [33,34]. Their use may explain some patients’ long-term persistence of respiratory function abnormalities.

This study has some limitations:

1. The analyzed studies are single-center studies, and severe/critical patients constitute a small group;
2. The inability to perform PFTs in the acute period makes assessing the degree of restrictive and obstructive respiratory disorders impossible;
3. According to the guidelines in force at the time, different methods of treating patients in different categories of disease severity were used;
4. Extended observation for several years using currently available and new diagnostic methods should be carried out.

## 5. Conclusions

1. Persistent respiratory symptoms after COVID-19, in some people, can result from fibrotic lung parenchyma changes, not visible in CT images.
2. The small airway disease after COVID-19 may result from post-infectious stenotic bronchitis due to persistent inflammation, even in mild/moderate COVID-19 patients.

## Data Availability

Data are unavailable due to privacy restrictions.
